# Use of sugar in coffee and tea and long-term risk of mortality in older adult Danish men: 32 years of follow-up from a prospective cohort study

**DOI:** 10.1371/journal.pone.0292882

**Published:** 2023-10-18

**Authors:** Roderick W. Treskes, Johan Clausen, Jacob L. Marott, Gorm B. Jensen, Andreas Holtermann, Finn Gyntelberg, Magnus T. Jensen

**Affiliations:** 1 Leiden University Medical Center, Department of Cardiology, Leiden, the Netherlands; 2 Steno Diabetes Center Copenhagen, Herlev, Denmark; 3 The Copenhagen Male Study, Copenhagen, Denmark; 4 The National Research Centre for the Working Environment, Copenhagen, Denmark; 5 William Harvey Research Institute, NIHR Bart’s Biomedical Research Centre, Queen Mary University of London, London, United Kingdom; Brigham and Women’s Hospital, UNITED STATES

## Abstract

**Background:**

Tea and coffee are the most consumed beverages worldwide and very often sweetened with sugar. However, the association between the use of sugar in tea or coffee and adverse events is currently unclear.

**Objectives:**

To investigate the association between the addition of sugar to coffee or tea, and the risk of all-cause mortality, cardiovascular mortality, cancer mortality and incident diabetes mellitus.

**Methods:**

Participants from the prospective Copenhagen Male Study, included from 1985 to 1986, without cardiovascular disease, cancer or diabetes mellitus at inclusion, who reported regular coffee or tea consumption were included. Self-reported number of cups of coffee and tea and use of sugar were derived from the study questionnaires. Quantity of sugar use was not reported. Primary outcome was all-cause mortality and secondary endpoints were cardiovascular mortality, cancer mortality and incident diabetes mellitus, all assessed through the Danish national registries. The association between adding sugar and all-cause mortality was analyzed by Cox regression analysis. Age, smoking status, daily alcohol intake, systolic blood pressure, body mass index, number of cups of coffee and/or tea consumed per day and socioeconomic status were included as covariates. Vital status of patients up and until 22.03.2017 was assessed. Sugar could be added to either coffee, tea or both.

**Results:**

In total, 2923 men (mean age at inclusion: 63±5 years) were included, of which 1007 (34.5%) added sugar. In 32 years of follow-up, 2581 participants (88.3%) died, 1677 in the non-sugar group (87.5%) versus 904 in the sugar group (89.9%). Hazard ratio of the sugar group compared to the non-sugar group was 1.06 (95% CI 0.98;1.16) for all-cause mortality. An interaction term between number of cups of coffee and/or tea per day and adding sugar was 0.99 (0.96;1.01). A subgroup analysis of coffee-only drinkers showed a hazard ratio of 1.11 (0.99;1.26). The interaction term was 0.98 (0.94;1.02). Hazard ratios for the sugar group compared to the non-sugar group were 1.11 (95% CI 0.97;1.26) for cardiovascular disease mortality, 1.01 (95% CI 0.87;1.17) for cancer mortality and 1.04 (95% CI 0.79;1.36) for incident diabetes mellitus.

**Conclusion:**

In the present population of Danish men, use of sugar in tea and/or coffee was not significantly associated with increased risk of mortality or incident diabetes.

## 1 Introduction

Coffee and tea are among the most consumed nonalcoholic hot beverages worldwide. Adding sugar to coffee or tea is common practice worldwide [1]. The association between coffee and tea consumption and all-cause mortality has been subject of various research projects [2–6]. Some large observational studies have found a U-shape curved association between the amount of coffee consumed per day and all-cause mortality [3, 5, 7]. Tea has been found to be associated with a decreased risk of all-cause mortality as well [6].

Sugar-sweetened beverages, such as sodas and fruit juices, have shown a dose-response association with adverse health outcomes [8]. Studies have shown an increased risk of obesity, dyslipidemia and type II diabetes mellitus in participants with high intakes of sugar-sweetened beverages [9–11]. Moreover, a recent study found that each additional 12-oz of sugary beverages increased the risk of all-cause mortality [8]. Several studies, moreover, have found an association between not only cardiovascular disease but also cancer mortality [12–14].

Nevertheless, the association between adding sugar to coffee and tea and adverse health outcomes has been less well studied. The amount of sugar consumed in traditionally consumed tea and coffee is usually smaller compared to other sugar-sweetened drinks [15–18]. Adding sugar to tea and coffee has previously been shown to be associated with higher inflammatory markers and higher insulin resistance. However, this paper did not include data on the difference in occurrence of major adverse cardiac events [1].

The primary purpose of this study was to investigate the association between adding sugar to coffee and/or tea and all-cause mortality in 32 years of follow-up from a prospective cohort of older adult Danish men. Secondarily, the study aimed to investigate the association between adding sugar to tea and/or coffee and cardiovascular mortality, cancer mortality and incident diabetes.

## 2 Methods

### 2.1 Study population

Details of the *Copenhagen Male Study (CMS)* have been published elsewhere [19]. The cohort was established in 1970–71 where 5245 males aged 40–59 were recruited from workplaces in Copenhagen. Initial examination included a physical examination and assessment of cardiorespiratory fitness. In addition, participants were interviewed by a physician, and a questionnaire on cardiovascular risk factors was completed. The participants were subdivided into 3 socioeconomic classes based on level of education and current occupation [20]. All participants were invited for a second follow up visit between 1985–1986 for an extensive clinical examination including measurement of diastolic and systolic blood pressure, weight and height. In addition, an extensive questionnaire about former and current diseases and lifestyle factors was completed. The questionnaire included detailed information about prior cardiovascular disease, diabetes, cancer, smoking habits and alcohol consumption. Details of the 1985–86 questionnaire have been published elsewhere [21]. Furthermore, the participants were asked to estimate their daily intake of coffee and tea (cups/day) and whether participants added sugar to their coffee (yes/no) or tea (yes/no). In the present study, we assumed that the participants added sugar to all cups of coffee and/or tea. Smoking status (present, former or never), daily alcohol intake, systolic blood pressure and body mass index (BMI) were assessed from the 1985/86 follow up questionnaire. Social group was divided in three categories: high, medium and low, based on a previous classification system by Svalastoga et al. [22]. Patients with an academic degree, higher education or were self-employed with >5 employees were classified as “high”. White collar workers or skilled blue-collar workers were classified as “medium”, while unskilled blue- collar workers were classified as “low”.

**Fig 1:**
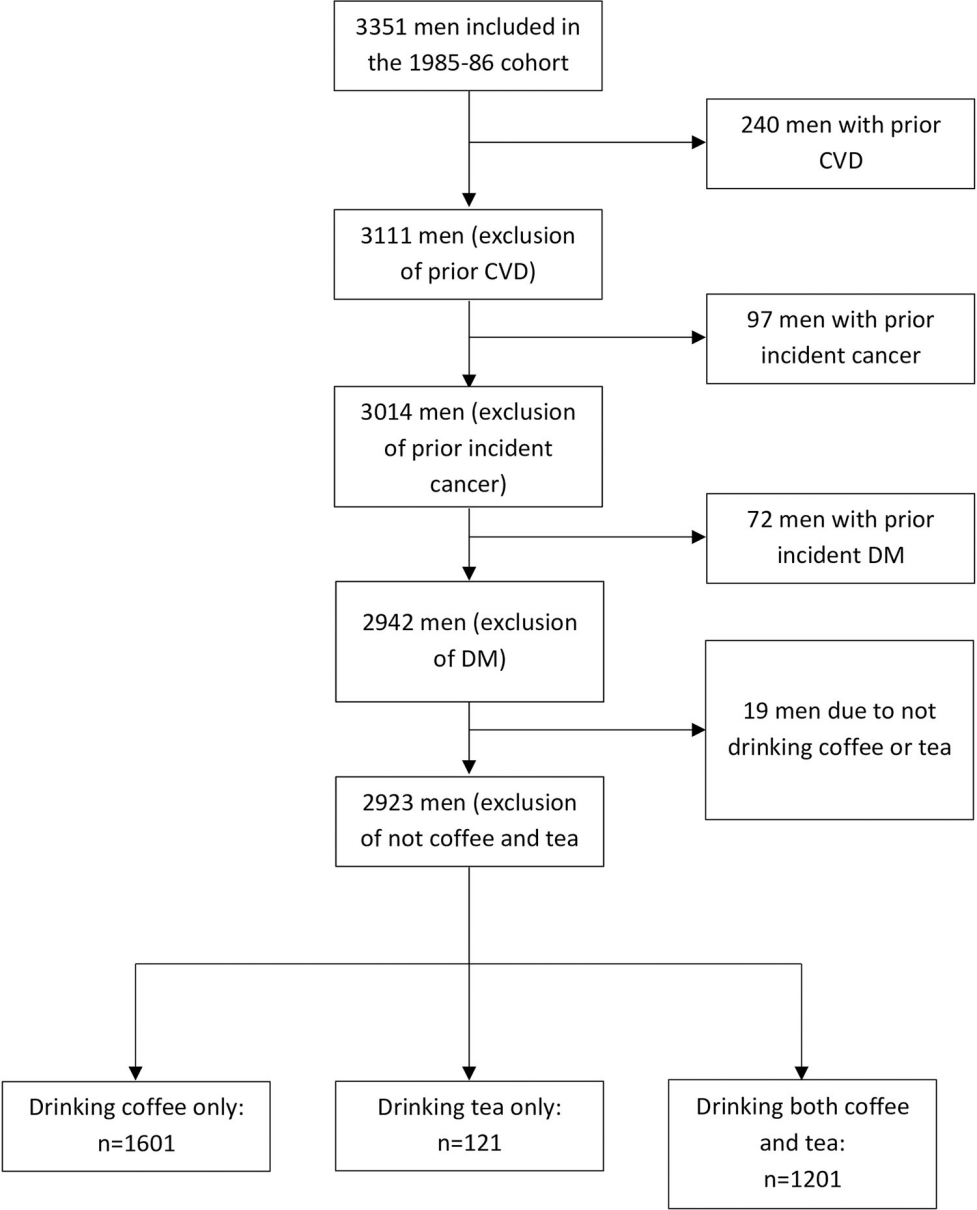
Flowchart of number of subjects included per category.

We excluded participants who answered yes to having diabetes (N = 76) or cardiovascular disease (N = 240) at inclusion in either the 1970 or 1985/86 questionnaire and participants who did not drink either coffee or tea (N = 20) (Fig 1). Of 3351 men who participated in the second follow up, 2923 men were included in the present study.

### 2.2 Patient and public involvement

As the majority of the CMS study participants was middle aged and included in 1970, consequently, the majority of the study participants was deceased at the time of the present analysis. However, during recruitment, patients were informed about the purpose of the study and kept up to date on its results.

### 2.3 Endpoint assessment

All-cause mortality was used as the primary endpoint by extracting vital status per 22.03.2017 from the national Danish Civil Registration System. Secondary endpoints were cardiovascular specific mortality (ICD-10: I00-I99 & ICD-8: 390–458), cancer specific mortality (ICD-10: C00-C97 & ICD-8: 140–207) and incident diabetes (ICD10: E10-E14 & ICD-8: 250) which were extracted from the national Danish Cause of Death Registry and the national Danish Patient Registry per 09.03.2017. In both registries, outcomes in >99% of patients could be assessed.

### 2.4 Statistical analysis

Stata version 12.1 (Stata-Corp, College Station, Texas) and R version 3.2.0 (R Foundation for Statistical Computing, Vienna, Austria) were used to perform statistical analyses. Subjects with missing values in any of the covariates included in the multivariable regression analyses were excluded (N = 133). Baseline characteristics are presented for the sugar group and non-sugar group. Statistical significance between both groups was calculated using a Pearson’s Chi-Squared test for categorical variables and a student’s T-test for continuous variables. A log-rank test was performed to compare the distributions of time to event occurrence in two groups. A P-value of <0.05 was considered statistically significant. The association between sugar use and the primary and secondary outcomes was analyzed using Cox proportional hazards regression models. Overall sugar addition to either tea or coffee (yes/no) was analyzed for all participants as the primary exposure. To eliminate the potential confounding effect of the difference in substance between coffee and tea, we performed a subgroup analysis in participants who drank coffee, but did not drink tea (“only coffee-drinkers”, N = 1653). As such, the regression analysis was repeated using only coffee-drinkers (N = 1653). Due to small numbers, a subgroup analysis in the tea only group (n = 126) was not feasible. Age, smoking status (never, former, current), body mass index, social group in 1970/1971 (high, medium, low), systolic blood pressure, units of alcohol and number of cups of coffee and/or tea consumed per day were included in the multivariable model as potential confounders. It was chosen not to adjust for multiple testing, in order not to increase the chance of making a type II error. An interaction term between number of cups of coffee and/or tea per day was introduced to investigate whether a dose-response relationship was present. A stratification analysis was performed for the association between sugar use and incident diabetes mellitus in the coffee-only subgroup. In order to perform this analysis, the group was stratified in two groups, participants who drank less than-, or equal to the median cups (n = 6) of coffee per day and participants who drank more.

### 2.5 Ethical approval

No medical ethics committee existed at the time of the original (1970–1971) protocol for the Copenhagen Male Study. The second cohort (1985–1986) was approved by a medical ethics committee, an ethics ID is not available since documentation is only kept for a limited time. The current study is approved by the steering committee of the Copenhagen Male Study. Participants consented in 1970–1971 orally for participation. No written informed consent was obtained. This was not deemed necessary by the medical ethics committee in 1985. The Copenhagen Male Study did not include minors.

## 3 Results

### 3.1 Population characteristics

In total 2923 participants of the 1985–1986 cohort of the Copenhagen Male Study were included in the study. These participants had a mean age of 62.7 years, with a standard deviation of 5.1 years. There were statistically significant differences in age, BMI, systolic BP and smoking status between the sugar group and non-sugar group. All baseline characteristics are listed in Table 1. In total, 133 participants had one or more missing values in the covariates. As such, 2790 patients were included in the multivariable analysis.

**Table 1:** Baseline characteristics of both groups.

	Total	Non-sugar group	Sugar group	P-value
N	2923	1916	1007	
Age in 1985 (mean±sd)	62.7±5.1	62.4±5.0	63.2±5.3	<0.001
BMI (mean±sd)	25.7±3.4	26.0±3.4	25.0±3.1	<0.001
Systolic BP (mean±sd)	121.1±15.9	121.8±15.9	119.8±15.9	0.002
Smoking in 1985				
• Present (yes/no)	1626 (55.2)	968 (50.5)	658 (65.3)	<0.001
• Former (yes/no)	939 (32.1)	684 (35.7)	255 (25.3)	
• Never (yes/no)	330 (11.3)	246 (12.8)	84 (8.3)	
• Missing	28 (1.0)	18 (0.9)	10 (1.0)	
Alcohol consumption				
• 0	301 (10.3)	180 (9.4)	121 (12.0)	0.077
• 1–2	1404 (48.0)	921 (48.1)	483 (48.0)	
• 3–5	981 (33.6)	647 (33.8)	334 (33.2)	
• >5	228 (7.8)	163 (8.5)	65 (6.5)	
• Missing	9 (0.3)	5 (0.3)	4 (0.4)	
Social economic class				0.094
• 1	561 (19.2)	386 (20.1)	175 (17.4)	
• 2	878 (30.0)	588 (30.7)	290 (28.8)	
• 3	1472 (50.4)	935 (48.8)	537 (53.3)	
• Missing	12 (0.4)	7 (0.4)	5 (0.5)	
Drinking only coffee	1601 (54.8)	1153 (60.2)	448 (44.5)	
Drinking tea	1322 (45.2)	763 (39.8)	559 (55.5)	<0.001
All-cause deaths (in 2017)	2581 (88.3)	1677 (87.5)	904 (89.9)	<0.001*
Cardiovascular deaths (in 2017)	1061 (36.3)	562 (35.3)	385 (38.2)	0.002*
Cancer deaths (in 2017)	859 (29.4)	562 (29.3)	297 (29.5)	0.102*
Incident diabetes mellitus (in 2017)	271 (9.3)	189 (9.9)	82 (8.1)	0.631*

*Log-rank test

** Statistical significance between both groups for continuous variables was calculated using a student’s t-test, for for endpoint percentageswith a Log-Rank test and categorical variables with a Pearson’s Chi-Squared Test. A P-value of <0.05 was considered statistically significant.

### 3.2 All-cause mortality

During 32 years of follow-up, 2581 participants (88.3%) died. In the non-sugar group, 1677 participants (87.5%) died, while in the sugar group 904 participants (89.9%) died (p<0.001). The hazard ratio of the sugar group compared to the non-sugar group was 1.06 (95% CI 0.98;1.6). An interaction term between number of cups of coffee and/or tea per day and adding sugar to tea and/or coffee was 0.99 (0.96:1.01), which suggest no interaction between adding sugar and number of cups of coffee in relation to all-cause mortality.

**Fig 2:**
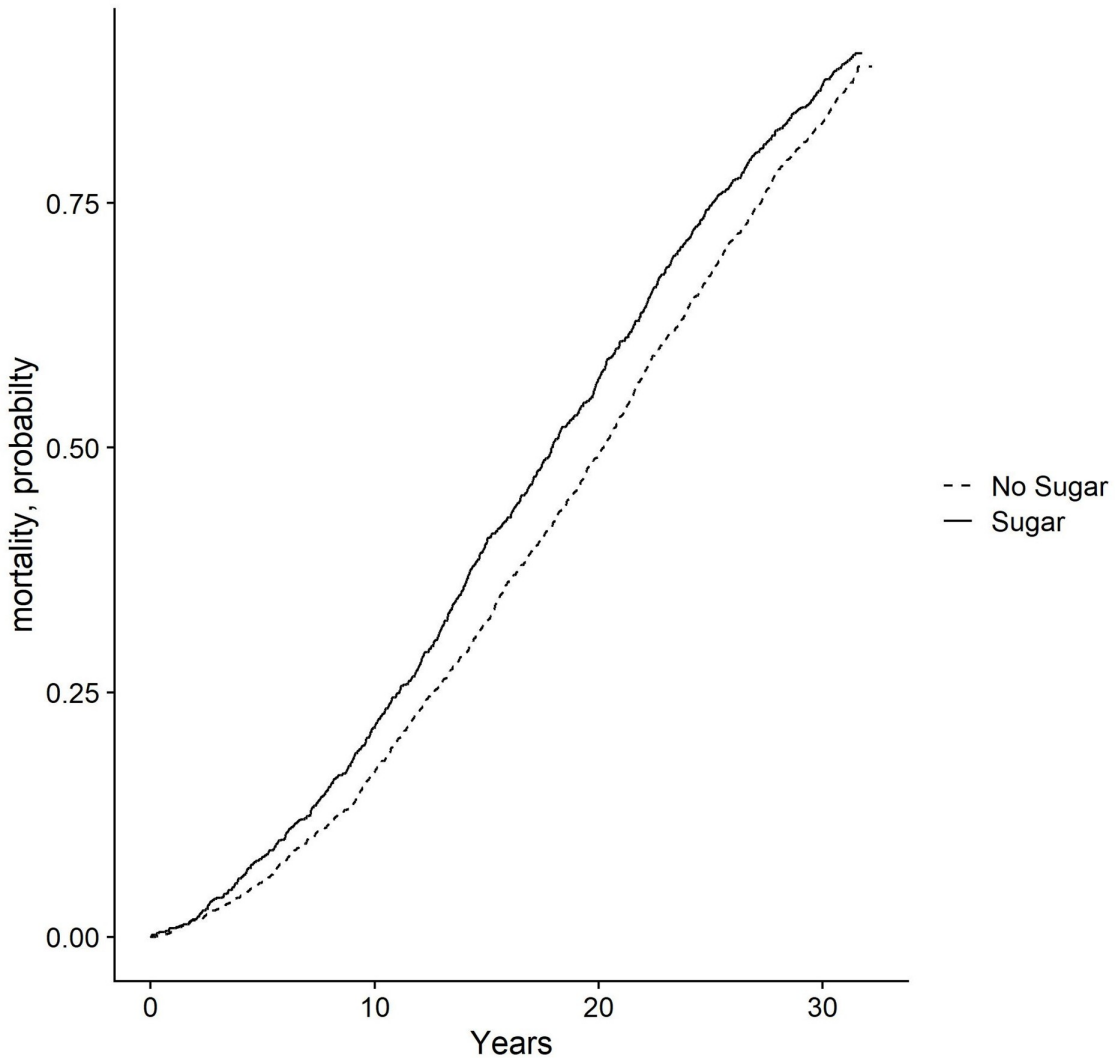
Cumulative incidence of all-cause mortality–stratified in sugar/no sugar group from the univariate analysis.

A subgroup analysis of participants who responded via the intake questionnaire that they drank coffee but did not drink tea (coffee-only drinkers), showed a similar association. Cox-regression analysis showed a hazard ratio for the sugar group compared to the non-sugar group of 1.11 (0.99:1.26). The interaction term between number of cups of coffee and/or tea per day and adding sugar to tea and/or coffee was 0.98 (0.94:1.02). All associations were non-significant. Results are given in Table 2. The cumulative incidence of all-cause mortality for both groups is plotted in Fig 2.

**Table 2:** Multivariable adjusted hazard ratios for the sugar group compared to the non-sugar group.

ALL-cause mortality			
Population	Exposure	Hazard ratio	P-value
All participants N = 2790	Sugar (y/n)	1.06 (0.98;1.16)	0.16
	Interaction term (sugar (y/n): cups of hot beverage)	0.99 (0.96;1.01)	0.36
Coffee-only drinkers N = 1528	Sugar (y/n)	1.11 (0.99;1.26)	0.08
	Interaction term (sugar (y/n): cups of hot beverage)	0.98 (0.94;1.02)	0.35

Hazard ratio (HR) of the sugar group compared to the non-sugar group, after correcting for the confounders age (in years), smoking status (never, former, current), body mass index (kg*m-2), social group in 1970/1971 (1, 2, 3), systolic blood pressure (mmHg), units of alcohol consumed (number) and number of cups of hot beverages per day. An interaction term between sugar intake (yes/no) and daily consumed cups of hot beverage is subsequently introduced. Results are shown for all study participants and for coffee- only drinkers as a subgroup.

### 3.3 Secondary endpoints

#### 3.3.1 Cardiovascular disease

During follow-up, 676 participants (35.3%) died of cardiovascular disease in the non-sugar group against 385 participants (38.2%) in the sugar group (p = 0.002). The hazard ratio for the sugar group compared to the non-sugar group was 1.11 (95% CI 0.97;1.26). The interaction term for cups of hot beverage and adding sugar (yes/no) was 0.99 (0.95;1.03). When analyzing coffee-only drinkers, the hazard ratio was 1.10 (0.91;1.33). The interaction term was 0.95 (0.89;1.01).

#### 3.3.2 Cancer

During follow-up, 859 participants (29.4%) died of cancer. In the non-sugar group, 562 participants (29.3%) died of cancer against 297 (29.5%) in the sugar group (p = 0.102). The hazard ratio for the sugar group compared to the non-sugar group was 1.00 (95% CI 0.87;1.17). The interaction term for cups of hot beverage and adding sugar (yes/no) was 0.99 (0.94;1.03). When analyzing coffee-only drinkers, the hazard ratio was 1.14 (0.93;1.39). The interaction term was 1.00 (0.94;1.06).

#### 3.3.3 Incident diabetes mellitus

During follow-up, 271 participants (9.3%) developed diabetes mellitus. In the non-sugar group, 189 participants (9.9%) developed diabetes mellitus against 82 participants (8.1%) in the sugar group (p = 0.631). The hazard ratio was 1.04 (95% CI 0.79;1.36). The interaction term for cups of hot beverage and adding sugar (yes/no) was 0.95 (0.86;1.04). When analyzing coffee-only drinkers, the hazard ratio was 0.91 (0.60;1.38). The interaction term was 0.82 (0.69;0.96).

## 4 Discussion

In this study, 2923 men free of prior cardiovascular disease, cancer and diabetes mellitus were followed for 32 years. At baseline, they indicated whether they drank coffee and tea and whether they added sugar to their coffee or tea. Outcomes of interest were all-cause mortality, cardiovascular mortality, cancer mortality and incident diabetes mellitus. Important findings of this study were that, when correcting for important confounders, there was no statistically significant association between the use of sugar in coffee and tea and all-cause mortality, cardiovascular mortality, cancer mortality or incident diabetes mellitus.

### 4.1 Sugar in relation to all-cause mortality

Literature on the association between adding sugar to coffee and/or tea and mortality is scarce. The finding of the present study are in line with a recently published study analyzing UK Biobank data [23]. This study investigated 186 811 UK Biobank participants. They investigated their intake of free sugars and correlated this to mortality. The study distinguished multiple sources of free sugars and found that the use of sugar in coffee and/or tea was not associated with an increased risk in mortality [23]. This study did not divide mortality into cancer and/or cardiovascular mortality. One study investigated the association between sugar in solids and liquids and markers of metabolic risk (notably serum Hemoglobin A1c, HbA1c, serum C-reactive protein, Homeostasis Model Assessment of Insulin Resistance, HOMA-IR, and the metabolic risk z-score). It showed that sugar added to coffee, tea and/or cereal was significantly associated with elevated HbA1c, CRP, HOMA-IR and metabolic risk score. In the same study, sugars from cakes, biscuits and confectionaries, as well as sugars from juice drinks, were not associated with elevated markers of metabolic risks. This study unfortunately did not include clinical events, leaving the clinical significance of these elevated risk scores somewhat unclear [1].

### 4.2 Possible explanation for the findings

The finding that adding sugar to coffee and tea does not significantly increase risk of all-cause mortality may seem counter intuitive. Previous research has indicated that high daily intake of sugar (especially from sugar-sweetened beverages) is associated with increased chances of cardiovascular adverse events and all-cause mortality [10, 11, 24]. First, the quantity of sugar in tea or coffee might play a role in this phenomenon. As Gyntelberg et al. [25] note, the average amount of sugar added to a cup of coffee or tea is 5 grams (two lumps of 2.5 gram), while an average can of sugar-sweetened beverage contains 25 grams of sugar. As there is a clear dose-response relationship between sugar intake and mortality, [26] the amount of ingested sugar via coffee and/or tea might simply be too low to substantially affect life expectancy and risk of developing diabetes.

Second, it might be that, had the sample size of this study been larger, the observed hazard ratios might have reached statistical significance. Nevertheless, with approximately 3000 participants, this is already a relatively large study. It has been demonstrated that high sugar intake per day is strongly associated with all-cause mortality. As such, had it been the case that daily intake of small amount of sugar induced a need for more sugar, it would have been expected that there was a significant difference in all-cause mortality between the group who added sugar to their coffee and the group who didn’t. In this line, it is also important to note that the data described in this paper, essentially reflect traditional coffee drinking (filtered black coffee). Major industries selling new types of coffee, such as caramel Frappuccino, did not reach Denmark until 2005–2008 [27]. Therefore, the results found in this study can only be extrapolated to individuals drinking coffee and tea in the traditional way.

### 4.3 Strengths and limitations

The study has several strengths: it is a study with 32-years of follow-up, well powered with almost 3000 men, almost complete follow-up with data being available for >99% of participants. Due to its long duration of follow-up and high incidence of the primary and secondary outcomes, reliable analyses could be performed.

The Copenhagen Male Study is an observational cohort study and has the limitations associated with not being a randomized controlled trial. One recent study, nevertheless, demonstrated that findings from observational studies in nutrition research are generally in line with findings from randomized controlled trials [28]. As such, given the prospective design, the long duration of follow-up and the low percentages of loss-to-follow-up, the results of this study are likely to be robust. A couple of specific limitations have to be kept in mind when analyzing the data: first, data on coffee, tea and sugar intake is self-reported. Therefore, the actual intake might deviate from the reported intake. Secondly, intake of coffee and/or teas and added sugar was assessed at a single time point. It was assumed that intake remained stable over the years. In reality, the amount of sugar added to each cup of coffee/tea or the number of cups per day they were adding it to might have decreased significantly over time in the sugar use group, which might have diminished the difference in exposure this study is trying to correlate to outcomes. It was also assumed that patients added sugar in moderation which is the traditional way of sweetening tea and coffee (e.g. one to two sugar cubes per cup of coffee and/or tea.). Thirdly, the consumer habits and findings in the present population of Scandinavian men included in 1985–86 may not be representative of other, contemporary populations. It is important to note that public attention for healthy diets has significantly increased over time. The addition of sugar to foods and beverages has several times been the subject of health campaigns during the last decades [29, 30]. Participants in the sugar group might have quitted using sugar in coffee and/or teas over time, limiting the number of participants exposed and reducing the difference in the exposure of interest in both groups [3, 5]. Fourthly, social economic status was assessed at a single time point as well and might have altered over the years. It has to be noted however that, on average, participants were 63 years old and as such, we hypothesize that the effect of SES on outcomes is limited if present. Fifthly, use of sugar in tea/coffee might have affected sugar intake from other product, such as sugar sweetened beverages. It remains to be investigated if intake from sugar in tea/coffee reduces intake from sugar sweetened beverage or influences other dietary options. This could explain the non-difference that was found in this study. The likelihood of a reduction remains to be investigated, as adding sugar to coffee/tea was associated with unhealthy behavior such as smoking. Lastly, this study was performed in older adult Danish men. As such, it remains to be investigated whether conclusions of this study can be extrapolated to other populations.

## 5 Conclusion

In the present population of Danish men, use of sugar in tea and/or coffee was not significantly associated with increased risk of mortality or incident diabetes.
